# The relationship between childhood traumatic life experiences and social anxiety disorders

**DOI:** 10.4102/sajpsychiatry.v32i0.2634

**Published:** 2026-07-30

**Authors:** Ayfer Ekim-Günaydın, Mazlum Çöpür

**Affiliations:** 1 Department of Nursing, Faculty of Health Sciences, Kırklareli University, Kırklareli, Turkey; 2 Department of Nursing, Faculty of Health Sciences, Istanbul Arel University, Istanbul, Turkey; 3 Department of Child and Adolescent Psychiatry, Faculty of Medicine, Kırklareli University, Kırklareli, Turkey

**Keywords:** social anxiety, childhood trauma, Liebowitz Social Anxiety Scale, adult, anxiety disorders, risk factors

## Abstract

**Background:**

Childhood traumatic experiences are known to have profound and lasting effects on mental health; however, there are limited data on the relationship between types of childhood trauma and social anxiety in non-clinical populations.

**Aim:**

This study aimed to explore the associations between types of childhood trauma and social anxiety symptoms in non-clinical young adults.

**Setting:**

The study was conducted at the Faculty of Health Sciences of a university in Istanbul, Turkey.

**Methods:**

This cross-sectional study included 486 university students. Sample size was determined via G*Power analysis to ensure statistical power. Participants completed the Liebowitz Social Anxiety Scale (LSAS) and the Childhood Trauma Questionnaire (CTQ) to assess childhood trauma types and social anxiety symptoms. Data were analysed using stepwise multiple regression.

**Results:**

The mean age of the participants was 21.8 ± 4.0 years, and 81% were female, with emotional abuse and neglect scoring highest in both genders. The sample’s LSAS total mean score was 63.87 ± 11.68. When the subscales were compared by gender, females had significantly higher mean fear and avoidance scores than males (*p* < 0.05). The results show that childhood abuse and neglect were significant predictors of social anxiety and explained 23% of the social anxiety variance.

**Conclusion:**

Childhood abuse and neglect are clearly associated with social anxiety symptoms. Interventions and treatments for individuals with social anxiety should incorporate a detailed assessment of childhood trauma history, with a particular focus on emotional abuse and neglect.

**Contribution:**

This study contributes to the literature by clarifying the associations between specific types of childhood trauma and social anxiety symptoms in a non-clinical young adult population.

## Introduction

Social anxiety disorder (SAD) is characterised by a persistent fear of interacting or performing in social situations due to concerns of embarrassment, humiliation or negative evaluation by others.^1^ Social anxiety disorder is the third most common psychiatric disorder, following major depression and alcohol dependence.^2^ According to epidemiologic studies, the lifetime prevalence of SAD is estimated to be between 2.8% and 13.0%.^3^ Although its exact aetiology remains unclear, multiple interrelated variables – such as genetic predisposition, temperament, family dynamics and environmental influences – contribute to its development.^4^ Most aetiological frameworks hypothesise that biological and psychological susceptibility traits interact and are subsequently exacerbated by a chronic cycle of negative thoughts, anxious feelings and behavioural avoidance.^2,3^ Regarding family-related factors, parental attitudes involving overprotectiveness, rejection, lack of interest and early adverse experiences frequently come to the fore as significant risk factors.^5,6^ According to the Diagnostic and Statistical Manual of Mental Disorders, Fifth Edition (DSM-5), characteristic symptoms of SAD include an intense fear of shame and negative evaluation when exposed to potential scrutiny by others.^1^ Social anxiety disorder is usually chronic, with an average age of onset ranging from 13 years to 15 years, significantly impairing academic, occupational and social functioning. Ultimately, SAD reduces overall quality of life while elevating the risk of substance dependence and suicidal ideation.^2,7^

Adverse experiences, such as childhood abuse and neglect, are considered critical components in the aetiology of SAD symptoms.^8^ Childhood traumatic experiences – defined as adverse life events occurring before the age of 18 years – function as profound risk factors that shape early behavioural patterns, rendering individuals more susceptible to psychological problems later in life.^9^ Extensive literature indicates that significant physical, emotional or sexual abuse during childhood routinely links to adult anxiety and depression.^10,11^ From a biological perspective, repeated exposure to severe stressors during childhood can permanently disrupt the architecture of the hypothalamic-pituitary-adrenal (HPA) axis, keeping the body in a perpetual state of vigilance via increased cortisol levels and elevating vulnerability to anxiety spectrum disorders in young adulthood.^12,13^ Indeed, contemporary studies report a substantial 15.5% – 32.8% increase in SAD symptoms among young adults who experienced early adversity.^14,15^

Theoretically, the distinct impact of emotional trauma on SAD symptoms can be conceptualised through cognitive-behavioural and interpersonal theories of psychopathology. Chronic emotional abuse and neglect directly disrupt early core relational schemas regarding self-worth and interpersonal safety.^16,17^ When primary caregivers repeatedly validate a child’s environment through emotional hostility or coldness, the child internalises cognitive models that depict the self as inherently unlovable and others as critical or rejecting.

This pervasive fear of negative evaluation forms the cognitive core of SAD.^10^ Consequently, emotional maltreatment is theoretically expected to be a more robust predictor of adult social anxiety than physical trauma, as it fundamentally attacks self-esteem and shapes chronic interpersonal vigilance.^16,18^

Although the overarching link between childhood trauma and social anxiety is established, more nuanced data are required to delineate how specific dimensions of trauma independently contribute to these symptoms.^11,19^

Understanding how distinct forms of childhood maltreatment manifest as adult social anxiety symptoms holds profound cross-cultural relevance, particularly for developing clinical screening frameworks in global contexts facing an alarmingly high prevalence of trauma, such as South Africa. Contemporary evidence from South African cohorts underscores that early-life trauma heavily impairs subsequent psychological functioning across both clinical and community populations.^8^ Investigating these pathways within a non-clinical sample can therefore provide critical baseline indicators for designing targeted, context-specific interventions in highly traumatised societies worldwide.^11^ The current study aimed to explore the associations between types of childhood trauma and SAD symptoms in non-clinical young adults. Identifying the relationship between SAD and types of childhood trauma has the potential to shed light on developmental risk factors for SAD.

## Research methods and design

This cross-sectional design study was conducted between March and September 2024.

### Study population and sampling strategy

The study population consisted of 1059 students studying at the Faculty of Health Sciences of a university in Istanbul. A convenience sampling strategy was utilised, and participants were recruited through face-to-face invitations during classroom visits, where they were provided with standardised information about the study’s purpose. The targeted number of participants was determined as 437 according to G*Power analysis (α = 0.05, 1-β = 0.80, *f*^2^ = 0.02). The study was completed with 486 participants who participated entirely on a voluntary basis. After the research was completed, a post-hoc analysis was conducted to determine the power of the study. With a Cohen *d* = 0.68 effect size, the power was found to be 90% at a 95% confidence level. The inclusion criteria for the study were as follows: Being at least 18 years old, being a registered student at the Faculty of Health Sciences, being able to understand Turkish and agreeing to participate voluntarily. Conversely, the exclusion criteria included: Having a self-reported or clinically diagnosed physical or psychological illness, providing incomplete questionnaires or refusing to sign the informed consent form.

### Instruments

#### Demographic information form

The form included 10 questions about socio-demographic characteristics such as gender, age, parental education and number of siblings.

#### Childhood trauma questionnaire-short form

The childhood trauma questionnaire-short form (CTQ-SF) is a retrospective self-report scale that assesses the history of childhood abuse and neglect. The scale is divided into five subscales: emotional abuse (EA), physical abuse (PA), sexual abuse (SA), emotional neglect (EN) and physical neglect (PN). The scale consists of 28 items, and item responses were scored between 1 and 5 (1 = ‘never true’ to 5 = ‘very often true’). The sum of the five sub-dimension scores gives the CTQ total score. Subdimension scores range from 5 (no neglect and abuse history) to 25 (very severe neglect and abuse history), and the total score ranges from 25 to 125. All items begin with the statement ‘When I was growing up …’. The scale developed by Bernstein et al.^20^ has been shown to have a generally good internal consistency with Cronbach’s α = 0.91. The Turkish adaptation of the scale was conducted by Tekin and Kırlıoğlu.^21^ Cronbach’s alpha of the Turkish form was found to be 0.97. Cronbach’s alpha values for the subdimensions were 0.87 for PN, 0.94 for EA sub-dimension, 0.83 for PA sub-dimension and 0.95 for the SA sub-dimension, respectively.

#### Liebowitz Social Anxiety Scale: Self-report version

The Liebowitz Social Anxiety Scale (LSAS) was developed to measure symptomatic distress and impairment caused by SAD. The LSAS is one of the most popular and widely used measures developed to measure social anxiety. The first rating is a measure of fear and ranges from 0 (none or very light) to 3 (severe). The second rating is a measure of avoidance and ranges from 0 (none or very rarely) to 3 (always). Each of the subscales consists of 24 items.

The time interval for rating each item is the participants’ experience in the past week. The total score is obtained by summing the scores obtained in both subscales. The scale was adapted into Turkish by Soykan and Ozguven.^22^ Cronbach’s alpha value for the total scale was 0.96, Cronbach’s alpha value for the fear sub-dimension was 0.96, for the avoidance sub-dimension was 0.95. In this study, the total scale had a Cronbach’s alpha value of 0.93. Cronbach alpha values for subdimensions were 0.91 for fear and 0.92 for the avoidance sub-dimension, respectively.

### Data collection

Prior to data collection, approval was obtained from the Ethics Committee and university administration. Following the official permissions, students were informed about the study and their questions were answered.

After that, students who signed the consent form and agreed to participate in the study were included in the study. The self-report questionnaires were completed in the classrooms in about 30 min. In the meantime, all participants were asked by the researcher to answer the questionnaires independently and not to discuss them with others. Completed questionnaires were delivered directly to the researchers.

### Data analysis

All statistical analyses were performed using Statistical Package for the Social Sciences (SPSS) version 23 (International Business Machines [IBM] SPSS Statistics, Armonk, NY, US).

Descriptive statistics, including frequencies, percentages, means and standard deviations, were calculated to summarise the characteristics of the sample. To evaluate the baseline associations between variables, separate Spearman correlation analyses were performed for male and female participants, examining the relationships between childhood trauma subtypes (CTQ) and the Liebowitz Social Anxiety Scale (LSAS) total and subscale scores. Subsequently, to evaluate the specific predictive value of childhood trauma subtypes on SAD and to test our hypotheses systematically, a series of stepwise multiple regression analyses was performed. In these multivariate models, the LSAS total score, fear subscale score and avoidance subscale score were treated as the dependent variables across separate equations, while the five specific childhood trauma subtypes (EA, EN, PA, PN and SA) were entered simultaneously as the independent variables using the stepwise selection method. Statistical significance level for all tests was determined as *p* < 0.05.

### Ethical considerations

The present study was approved by the Ethics Committee of our institution (approval number 050.06.04-255912) and conducted in accordance with the ethical standards of the World Medical Association Declaration of Helsinki. Participation was entirely voluntary, and written informed consent was obtained from all participants. To ensure strict confidentiality, all responses were recorded anonymously, with no personal identifiers linked to the data. Furthermore, the signed informed consent forms were stored separately from the data sheets to eliminate any risk of participant re-identification. Completed questionnaires were delivered directly to the researchers and stored in a secure, encrypted digital environment accessible only to the research team. Results were reported as aggregated data to prevent the identification of any individual participant.

## Results

### Sample characteristics

The sample consisted of 486 participants. Participants’ ages ranged from 18 to 34 years, with a mean age of 21.8 ± 4.0 years, and 81% (*n* = 393) were female and 19% (*n* = 93) were male. The mean number of siblings was 3.7 ± 2.7 (range = 1–12), and the majority were first (36.9%, *n* = 179) and second (28.6%, *n* = 139) children. Of the participants’ mothers, 31% (*n* = 150) were primary school graduates, 11.9% (*n* = 58) were middle school graduates, 19.0% (*n* = 92) were high school graduates and 36.9% (*n* = 180) were university graduates or above.

Of the fathers, 45.2% (*n* = 220) were primary school graduates, 14.3% (*n* = 70) were middle school graduates, 20.2% (*n* = 98) were high school graduates and 20.2% (*n* = 98) were university graduates or above. The mean number of people living in the same household was 2.7 ± 1.7 (range = 1–7). While 51.8% (*n* = 251) of the participants reported their economic status as medium, 48.2% (*n* = 235) reported it as low. Sixty-seven point nine percent (*n* = 330) of the participants used the internet between 1 h and 4 h a day, and 32.1% (*n* = 156) used the Internet for more than 5 h a day. Fifty-one point two percent (*n* = 248) of the participants were smokers. The socio-demographic characteristics of the study sample were summarised in Table 1.

**TABLE 1:** Sample characteristics (*N* = 486).

Variables	*n*	%
**Age (years)**		
18–25	432	89.0
26–33	40	8.2
> 34	14	2.8
**Gender**		
Female	393	81.0
Male	93	19.1
**Income**		
Low	231	47.6
Moderate	238	48.8
High	17	3.49
**Number of siblings**		
0–2	210	43.0
3–5	182	37.3
> 6	94	19.2
**Daily duration of internet use (hours)**		
0–4	330	67.9
> 5	156	32.1
**Alcohol use**		
Yes	161	33.1
No	325	66.8
**Smoking**		
Yes	176	36.3
No	310	63.7

### Childhood traumatic experiences

The mean CTQ total score was 55.57 ± 7.34 and was not significantly different between genders. Emotional abuse had the highest score among childhood traumas in both females and males (female = 15.83 ± 4.82; male = 12.06 ± 4.87), and the mean score of females was significantly higher than males (*p* < 0.001). The second highest score belonged to EN, and the third highest to PA subdimensions. The comparison of CTQ total and sub-dimension mean scores according to gender is shown in Table 2. When the CTQ total mean scores were compared according to the variables, there was a moderate negative significant relationship between the mother’s educational level and EN (*r* = −0.414; *p* = 0.004), EA (*r* = −0.859; *p* = 0.020) and PA (*r* = −0.594, *p* = 0.000). There was a moderate negative correlation between father’s education level and PA (*r* = −0.557, *p* < 0.001), PN (*r* = −0.495, *p* < 0.001), EN (*r* = −0.317, *p* = 0.004) and SA (*r* = 0.221, *p* = 0.043). There was a moderate positive correlation between the number of children in the family and EA (*r* = 0.682, *p* = 0.045) and SA (*r* = 0.598, *p* = 0.023) scores. Accordingly, an increasing number of children in the family increases the risk of emotional and SA. There was a negative moderate significant relationship between economic status and the mean scores of PA (*r* = −0.690, *p* < 0.001), EA (*r* = −0.490, *p* = 0.002) and SA (*r* = −0.509, *p* < 0.001).

**TABLE 2:** Childhood trauma questionnaire mean scores.

CTQ-SF subscales	Gender		*p*-value	Total
	Female	Male		
Emotional abuse	15.83 ± 4.82	12.06 ± 4.87	0.001*	13.41 ± 4.85
Physical abuse	9.80 ± 3.99	11.67 ± 4.54	0.039*	10.10 ± 4.12
Sexual abuse	7.23 ± 2.92	7.06 ± 2.95	0.453	7.20 ± 2.91
Physical neglect	9.69 ± 2.13	9.50 ± 2.42	0.416	9.65 ± 2.17
Emotional neglect	12.76 ± 3.28	11.03 ± 2.77	0.172*	11.53 ± 4.67
Total	55.67 ± 7.02	55.12 ± 8.79	0.231	55.57 ± 7.34

* , Statistical significance at *p* < 0.05.

### Social anxiety

The sample’s LSAS total mean score was 63.87 ± 11.68. The mean subdimension scores were 32.23 ± 6.49 for the fear sub-dimension and 31.64 ± 5.39 for the avoidance sub-dimension, respectively. Liebowitz Social Anxiety Scale total mean scores did not significantly differ by gender (females: 63.94 ± 15.93; males: 63.50 ± 14.65, *p* = 0.311).

However, in the comparison of the subdimensions of LSAS by gender, females had significantly higher mean fear scores (female = 32.58 ± 6.43; male = 30.15 ± 5.06, *p* = 0.020) and avoidance scores (female = 31.36 ± 5.40; male = 29.26 ± 4.71, *p* = 0.011) than males.

The correlations between the total and subdimensions of LSAS and CTQ were tested for both genders and the total sample (Table 3). Accordingly, there was a significant positive correlation between the LSAS total score and the CTQ total score for both males (*r* = 0.759, *p* = 0.001) and females (*r* = 0.396, *p* = 0.033). In both genders, a significant positive correlation was found between EA and the fear score (males: *p* = 0.007; females: *p* = 0.043). Additionally, there was a highly significant positive relationship between SA and the avoidance sub-dimension score in females. There was no significant difference in fear and avoidance behaviours according to age, number of siblings and parents’ education level (*p* > 0.05).

**TABLE 3:** Correlation between LSAS and childhood trauma questionnaire scores.

Childhood trauma questionnaire	Liebowitz Social Anxiety Scale: Self-Report Version (LSAS)											
	Fear				Avoidance				Total			
	Male		Female		Male		Female		Male		Female	
	*r*	*p*-value	*r*	*p*-value	*r*	*p*-value	*r*	*p*-value	*r*	*p*-value	*r*	*p*-value
Emotional abuse	0.689	0.007*	0.545	0.043*	0.249	0.372	0.006	0.961	0.806	0.029*	0.486	0.012*
Physical abuse	0.709	0.005*	0.683	0.079	−0.005	0.987	0.012	0.304	0.130	0.211	0.234	0.098
Physical neglect	0.637	0.124	0.387	0.038*	0.478	0.041*	0.079	0.523	0.593	0.161	0.571	< 0.001*
Emotional neglect	0.423	0.345	0.252	0.187	0.207	0.460	0.568	0.040*	0.797	0.003*	0.749	< 0.001*
Sexual abuse	0.810	< 0.001*	0.518	0.004*	0.458	0.086	0.821	0.006*	0.197	0.086	0.510	< 0.001*
Total	0.759	< 0.001*	0.396	0.033*	0.597	0.019*	0.744	0.040*	0.596	0.014*	0.815	0.033*

Note: *r* = Spearman’s correlation coefficients.

LSAS, Liebowitz Social Anxiety Scale.

* , *p* < 0.05.

### Regression analysis

In this study, stepwise multiple regression analyses were used to determine the predictors of SAD. In Model 1, where the LSAS total score was treated as the dependent variable, the regression model was statistically significant (*Adj-R*^2^ = 0.237, *F* = 2.828, *p* = 0.027). Among the independent variables, the CTQ total score (*β* = 1.298, *p* = 0.017), EA (*β* = 1.333, *p* < 0.001), SA (*β* = 1.908, *p* = 0.032), EN (*β* = 0.105, *p* = 0.016) and PN (*β* = 0.133, *p* = 0.026) exerted significant predictive effects, collectively accounting for 23.7% of the total variance in social anxiety symptoms (Table 4). Physical abuse did not emerge as a significant predictor for the total score (*p* = 0.212). In Model 2, which evaluated the LSAS fear sub-dimension as the dependent variable, the model demonstrated a strong explanatory capacity (*Adj-R^2^* = 0.317, *F* = 18.01, *p* < 0.001). Significant predictive effects on fear behaviours were observed for the CTQ total score (*β* = 0.588, *p* < 0.001), EA (*β* = 0.391, *p* = 0.018), SA (*β* = 0.644, *p* < 0.001) and EN (*β* = 0.290, *p* = 0.031). Conversely, PA (*p* = 0.494) and PN (*p* = 0.224) did not significantly predict fear symptoms. This model achieved an overall explanatory power of 31.7% for the fear dimension variance.

**TABLE 4:** Stepwise multiple regression results for childhood trauma and social anxiety dimensions.

Dependent variables	Independent variables	*R* ^2^	Adj-*R*^2^	RMSE	*F*	B	SE	95% CI		*β*	*t*-value	*p*-value
								Lower	Upper			
Fear	-	0.346	0.317	4.621	18.010	-	-	-	-	-	-	< 0.001*
	Total	-	-	-	-	0.804	0.190	0.419	1.190	0.588	4.244	< 0.001*
	Physical abuse	-	-	-	-	0.455	0.658	0.882	1.791	0.118	0.692	0.494
	Emotional abuse	-	-	-	-	1.955	0.789	0.351	3.559	0.391	2.477	0.018*
	Sexual abuse	-	-	-	-	2.662	0.542	1.561	3.763	0.644	4.912	< 0.001*
	Emotional neglect	-	-	-	-	1.814	1.025	0.669	3.897	0.290	1.769	0.031*
	Physical neglect	-	-	-	-	2.680	0.912	0.828	4.533	0.450	2.941	0.224
Avoidance	-	0.269	0.178	1.897	2.696	-	-	-	-	-	-	0.020*
	Total	-	-	-	-	0.149	0.171	0.191	0.490	0.096	0.871	0.034*
	Physical abuse	-	-	-	-	0.182	0.006	−1.274	0.098	1.320	3.761	0.038*
	Emotional abuse	-	-	-	-	0.263	0.472	6.764	1.201	0.062	0.556	0.020*
	Sexual abuse	-	-	-	-	0.238	0.260	−0.509	1.206	0.101	0.916	0.363
	Emotional neglect	-	-	-	-	0.695	0.052	−2.791	0.755	0.610	0.058	0.580
	Physical neglect	-	-	-	-	0.810	0.582	−0.347	1.967	0.153	1.393	0.167
Total	-	0.309	0.237	1.313	2.828	-	-	-	-	-	-	0.027*
	Total	-	-	-	-	0.552	0.397	1.086	2.072	1.298	0.309	0.017*
	Physical abuse	-	-	-	-	1.891	2.540	3.312	0.057	0.228	0.134	0.212
	Emotional abuse	-	-	-	-	0.682	0.549	2.876	0.897	1.333	2.432	< 0.001*
	Sexual abuse	-	-	-	-	1.543	1.000	1.976	0.652	1.908	0.765	0.032*
	Emotional neglect	-	-	-	-	1.025	1.849	4.434	3.128	0.105	0.353	0.016*
	Physical neglect	-	-	-	-	2.110	2.675	5.822	9.210	0.133	0.461	0.026*

Adj-*R*^2^, adjusted *R*-square; RMSE, root mean square error; SE, standard error; CI, confidence interval; *β*, standardised coefficients.

* , *p* < 0.05.

Finally, in Model 3, where the LSAS avoidance subscale served as the dependent variable, the regression model was statistically significant (*Adj-R^2^* = 0.178, *F* = 2.696, *p* = 0.020). The CTQ total score (*β* = 0.096, *p* = 0.034), PA (*β* = 1.320, *p* = 0.038) and EA (*β* = 0.062, *p* = 0.020) were identified as significant predictors, explaining 17.8% of the variance in avoidance behaviours (Table 4).

## Discussion

The current study investigated the relationship between childhood traumas and SAD in a population of university students. According to the results of the study, childhood traumas were a predictor of LSAS total score and sub-dimension scores (fear and avoidance). This result is consistent with previous studies suggesting a link between childhood traumas and SAD.^3,11,17^ Studies in non-clinical samples show that negative experiences such as childhood traumas are associated with depression, anxiety and emotional-behavioural disorders.^3,23,24^ Specifically, recent research in non-clinical samples underlines that early adverse experiences, particularly emotional maltreatment, significantly elevate the risk of developing general anxiety and depressive symptoms in young adulthood. For instance, these studies have demonstrated that individuals with a history of childhood EN exhibit higher levels of emotional dysregulation and severe behavioural avoidance in social settings, further validating the long-term psychological burden of non-clinical trauma.^25,26^ Although it has been shown that there is a strong link between child abuse and SAD, the results are limited in revealing the relationship between different types of abuse and SAD. According to the study results, EA was a stronger predictor of LSAS total and its subscales (fear and avoidance). This result suggests that parents’ emotional expressions towards their children may have long-term effects and contribute to SAD. Indeed, studies show that EA and neglect are at the forefront of anxiety disorders.^11^ For example, Gibb et al.^17^ showed that childhood EA is more strongly associated with SAD than PA or SA. Similarly, in the meta-analysis study of Liu et al.^23^ a stronger relationship between EA and neglect and SAD than other types of abuse is reported. Bandelow et al.^24^ reported that patients with SAD have negative parental rearing styles, such as inadequate affection and care, compared to controls, and that these patients have high sensitivity to rejection and criticism. Simon et al.^18^ reported that 56% of individuals with SAD confirmed a history of childhood exposure to EA and 39% to EN. However, most of these studies included clinical samples. Our study differed from these in that it showed results from a healthy sample.

Understanding these associations in non-clinical populations holds significant global relevance, particularly for countries with an exceptionally high prevalence of trauma, such as South Africa. For instance, contemporary South African research highlights that early traumatic life events heavily impact psychological functioning and quality of life across both clinical and community cohorts.^8^ Therefore, demonstrating that EA and neglect predict SAD symptoms even in healthy, non-clinical young adults underscores the urgent cross-cultural need for early screening and trauma-focused interventions in high-adversity environments worldwide.^9^ Emotional abuse is considered to be the most destructive form of abuse; its negative consequences can accompany children’s lifelong development.^26,27^ According to interpersonal theories, experiences during childhood form cognitive structures and relational schemas. Childhood trauma experiences negatively affect individuals’ self-perception. In particular, abuse of an emotional nature contributes to feelings of being unloved while also causing fear of interacting with others. The individual may develop a schema in which self-esteem and personal worth depend on the opinion of others.^28,29^ They may have lower self-esteem and be more self-critical, and this may make others’ criticism or negative evaluation more threatening for them.^30^

According to the study results, SA was a predictor of LSAS total and fear dimensions. A review of the literature indicates that the majority of studies investigating the effect of SA on anxiety disorders have predominantly focused on women with SAD. In these studies, it has been shown that the risk of SAD is higher in the population exposed to SA.^31^ Although many studies mention a stronger relationship between EA and neglect and SAD than physical and SA, according to our results, PA was a predictor of the avoidance subscale.^32^ Studies show different results regarding the relationship between sexual and PA and SAD. For example, Nanda et al.^33^ showed SA and PA to be predictors of social anxiety symptoms, while Hamilton et al.^29^ and Kircaburun et al.^30^ mentioned a stronger relationship between social anxiety symptoms and EA and neglect than other types of abuse. From a structural perspective, these different trauma subtypes map differentially onto the fear versus avoidance dimensions of SAD. Emotional and SA directly fuel the cognitive ‘fear’ sub-dimension by creating highly sensitive threat-monitoring schemas focused on immediate social threat, embarrassment and scrutiny.^16^ In contrast, our findings that PA uniquely predicts the ‘avoidance’ sub-dimension can be explained by behavioural learning principles. Physical violence involves overt, inescapable aversive stimuli; in young adulthood, the individual translates the coping mechanism of physical safety into generalised ‘behavioural avoidance’ to proactively escape any potential social confrontation or perceived hostility.^11^ The main reason for the different results may be the different characteristics of the sample group of the studies conducted on this subject. While the sample of most studies was a clinically diagnosed SAD group, the sample of very few studies was a non-clinical population. More study results with similar samples are needed to show the relationship between physical and SA and SAD.^34,35^

According to the study results, the LSAS total score did not differ significantly by gender. However, both the fear and avoidance sub-dimension scores were significantly higher in females than in males. Studies on the relationship between gender and SAD show mixed results. For example, in the study of Olivares-Olivares et al.,^36^ fear and avoidance scores were significantly higher in females than in males in the Spanish population. Caballo et al.^34^ reported that social anxiety scores were higher in females than in males in their study, but the result was not significant when the magnitude of the differences was examined. Asher et al.^35^ examined the existing literature in terms of many parameters, such as functionality, comorbidity, prognostic indicators, health service utilisation and oxytocin status, to determine the differences between genders and found that SAD affects both genders equally. In Dell’osso et al.’s^37^ study, females show higher rates of SAD symptoms. Caballo et al.’s^34^ study of large sample clinical participants from 18 countries shows that there are significant differences in SAD between males and females. However, most of these studies include clinical samples, highlighting the need for more data in non-clinical populations.

In our sample, EA yielded the highest score, followed by EN and PA. A similar result was shown in the study of Yang et al.^25^ conducted with a similar sample group in China, where EN followed by PA was reported as the most common childhood trauma. Studies on this issue show that EA and EN are common forms of childhood trauma.^33,35^

According to our findings, females reported higher exposure to EA than males, whereas males reported higher exposure to PA than females. Consistent with these results, Tang et al.^38^ demonstrated that males are more frequently exposed to PA. Similarly, Meng and D’Arcy^39^ reported that males experience more PA and violent behaviours, while females are more prone to SA. These variations underscore that cultural characteristics and societal differences in child-rearing attitudes play a crucial role in gender-based patterns of exposure to childhood trauma.^40^ The findings of this study highlight the need for routine clinical screenings for childhood trauma – especially EA and neglect – in patients with social anxiety. Both clinicians and patients often underestimate emotional trauma, perceiving it as less severe than PA or SA.^41,42^ Therefore, raising awareness about different trauma types and utilising trauma-informed screening tools is crucial to detect these subtle early adversities in psychiatric settings.

### Strengths and limitations

The main strength of this study is that it examines the associations between childhood traumatic experiences and SAD within a non-clinical population. Our findings contribute to a deeper understanding of the social and psychological mechanisms underlying early adversity. We accept that the present study has certain limitations. Firstly, the assessment of childhood trauma was based on participants’ retrospective reports, which may introduce recall bias regarding early life events. Secondly, this study relied entirely on self-report measures to assess both childhood trauma and SAD. Because self-reports are inherently subjective, participants may have over- or underestimated their experiences. Thirdly, the sample was drawn from a single Faculty of Health Sciences at a single university, and the participants were predominantly female, which restricts the generalisability of the findings to the broader young adult population. Fourthly, we did not assess the duration or severity of childhood trauma, meaning our results do not provide data on these specific dimensions of effect. Future multi-centre studies with a more balanced gender distribution are needed to enhance the generalisability of these associations.

## Conclusion

This study investigated the associations between childhood traumatic life experiences and SAD in a non-clinical sample. Findings indicated that childhood traumatic experiences were a significant predictor of SAD in young adulthood. The most common childhood traumatic experience was EA, followed by EN and PA. Regarding SAD subscores, fear and avoidance scores were significantly higher in females than in males. These noteworthy results suggest that childhood traumatic experiences should be carefully considered in the assessment of SAD. Furthermore, our findings serve as a reminder that it is crucial to assess the specific type of childhood trauma individuals have experienced when evaluating SAD. Despite the historical tendency in the anxiety literature to focus primarily on sexual and PA, our results add to the growing body of literature demonstrating that EA is heavily associated with SAD.

Childhood trauma can cause severe emotional pain and distress, often leading to lifelong damage to physical and mental health. Crucially, untreated early adversity and underdiagnosed SAD can potentially trigger a cascading chain of psychological challenges during young adulthood. When early social avoidance remains unrecognised during formative years, it may function as a developmental risk factor that elevates long-term vulnerability to secondary emotional or behavioural complications. To break this cycle of maltreatment, clinicians must remain aware of these associations, thoroughly investigate adverse childhood experiences and initiate trauma-focused therapeutic interventions when appropriate. Furthermore, educational institutions and health services should focus on the early recognition of childhood trauma, providing relevant education and supportive interventions to prevent the long-term development of severe SAD symptoms.
